# CompSafeNano project: NanoInformatics approaches for safe-by-design nanomaterials

**DOI:** 10.1016/j.csbj.2024.12.024

**Published:** 2024-12-25

**Authors:** Dimitrios Zouraris, Angelos Mavrogiorgis, Andreas Tsoumanis, Laura Aliisa Saarimäki, Giusy del Giudice, Antonio Federico, Angela Serra, Dario Greco, Ian Rouse, Julia Subbotina, Vladimir Lobaskin, Karolina Jagiello, Krzesimir Ciura, Beata Judzinska, Alicja Mikolajczyk, Anita Sosnowska, Tomasz Puzyn, Mary Gulumian, Victor Wepener, Diego S.T. Martinez, Romana Petry, Naouale El Yamani, Elise Rundén-Pran, Sivakumar Murugadoss, Sergey Shaposhnikov, Vasileios Minadakis, Periklis Tsiros, Harry Sarimveis, Eleonora Marta Longhin, Tanima SenGupta, Ann-Karin Hardie Olsen, Viera Skakalova, Peter Hutar, Maria Dusinska, Anastasios G. Papadiamantis, L. Cristiana Gheorghe, Katie Reilly, Emilie Brun, Sami Ullah, Sebastien Cambier, Tommaso Serchi, Kaido Tämm, Candida Lorusso, Francesco Dondero, Evangelos Melagrakis, Muhammad Moazam Fraz, Georgia Melagraki, Iseult Lynch, Antreas Afantitis

**Affiliations:** a NovaMechanics Ltd, Nicosia 1070, Cyprus; b Entelos Institute, Larnaca 6059, Cyprus; c NovaMechanics MIKE, Piraeus 18545, Greece; d Finnish Hub for Development and Validation of Integrated Approaches (FHAIVE), Faculty of Medicine and Health Technology, Tampere University, Tampere 33520 Finland; e School of Physics, University College Dublin, Belfield, Dublin, Ireland; f QSAR Lab, Trzy Lipy 3, Gdańsk 80-172, Poland; g University of Gdansk, Faculty of Chemistry, Wita Stwosza 63, Gdansk 80-308, Poland; h Water Research Group, School of Biological Sciences, North-West University, Potchefstroom, North-West Province, South Africa; i Brazilian Nanotechnology National Laboratory (LNNano), Brazilian Center for Research in Energy and Materials (CNPEM), Campinas, Sao Paulo, Brazil; j Department of Environmental Chemistry and Health, Climate and Environmental Research Institute-NILU, Kjeller 2007, Norway; k NorGenoTech, Oslo, Norway; l School of Chemical Engineering, National Technical University of Athens, Heroon Polytechniou 9, Zografou 15780, Greece; m Danubia Nanotech, Bratislava, Slovakia; n School of Geography, Earth and Environmental Sciences, University of Birmingham, Edgbaston, Birmingham B15 2TT, United Kingdom; o Environmental Health research group, Luxembourg Institute of Science and Technology, 41 rue du Brill, Belvaux L4422, Luxembourg; p Institute of Chemistry, University of Tartu, Ravila 14A, Tartu 50411, Estonia; q Department of Science and Technological Innovation, Università del Piemonte Orientale, Viale Michel 11, Alessandria 15121, Italy; r Calculus IKE, Pireaus, Greece; s National University of Sciences and Technology (NUST), Islamabad, Pakistan; t Division of Physical Sciences and Applications, Hellenic Military Academy, Vari, Greece

**Keywords:** Computational approaches, nanomaterials safety, cloud platform, biomolecule interactions, nanoinformatics

## Abstract

The CompSafeNano project, a Research and Innovation Staff Exchange (RISE) project funded under the European Union's Horizon 2020 program, aims to advance the safety and innovation potential of nanomaterials (NMs) by integrating cutting-edge nanoinformatics, computational modelling, and predictive toxicology to enable design of safer NMs at the earliest stage of materials development. The project leverages Safe-by-Design (SbD) principles to ensure the development of inherently safer NMs, enhancing both regulatory compliance and international collaboration. By building on established nanoinformatics frameworks, such as those developed in the H2020-funded projects NanoSolveIT and NanoCommons, CompSafeNano addresses critical challenges in nanosafety through development and integration of innovative methodologies, including advanced *in vitro* models, *in silico* approaches including machine learning (ML) and artificial intelligence (AI)-driven predictive models and 1st-principles computational modelling of NMs properties, interactions and effects on living systems. Significant progress has been made in generating atomistic and quantum-mechanical descriptors for various NMs, evaluating their interactions with biological systems (from small molecules or metabolites, to proteins, cells, organisms, animals, humans and ecosystems), and in developing predictive models for NMs risk assessment. The CompSafeNano project has also focused on implementing and further standardising data reporting templates and enhancing data management practices, ensuring adherence to the FAIR (Findable, Accessible, Interoperable, Reusable) data principles. Despite challenges, such as limited regulatory acceptance of New Approach Methodologies (NAMs) currently, which has implications for predictive nanosafety assessment, CompSafeNano has successfully developed tools and models that are integral to the safety evaluation of NMs, and that enable the extensive datasets on NMs safety to be utilised for the re-design of NMs that are inherently safer, including through prediction of the acquired biomolecule coronas which provide the biological or environmental identities to NMs, promoting their sustainable use in diverse applications. Future efforts will concentrate on further refining these models, expanding the NanoPharos Database, and working with regulatory stakeholders thereby fostering the widespread adoption of SbD practices across the nanotechnology sector. CompSafeNano's integrative approach, multidisciplinary collaboration and extensive stakeholder engagement, position the project as a critical driver of innovation in NMs SbD methodologies and in the development and implementation of computational nanosafety.

## Graphical Abstract

**Figure:**
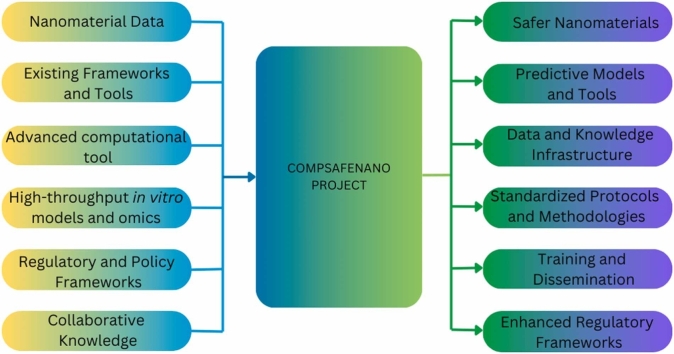

## 1 Introduction

Nanotechnology has enabled the discovery and development of a wide range of innovative nanomaterials (NMs) that are applied in all industry sectors, laying the groundwork for the emergence of Advanced and Smart Materials. These materials are revolutionising a broad spectrum of industries, ranging from consumer products to aerospace, personalised medicine, and precision agriculture. The intrinsic physicochemical properties at the nanoscale, which differ significantly from their bulk counterparts, have led to the integration of NMs into an expanding portfolio of commercial applications [1], [2], [3]. However, the introduction of novel properties by NMs also brings inherent challenges, particularly in terms of potential health and environmental impacts [4], [5], [6]. Assessment of the potential risks associated with NMs should progress concurrently with the exploration and exploitation of their benefits, reflecting the principles of responsible innovation. Like most chemicals, the full spectrum of potential biological effects of many NMs remains under-explored due to the absence of long-term data. Moreover, traditional experimental assessments of these materials (and all chemicals) are not only costly and time-consuming but also involve animal testing, which raises ethical concerns [7]. There is also a knowledge gap whereby findings regarding the NMs properties driving their reactivity and toxicity are not being effectively fed back into the re-design of NMs, via the process of Safe-by-Design (SbD). This is, in part, due to the breadth of nanosafety data and the challenges for materials developers to keep up with the evolving science in parallel with their business challenges.

Hazard and risk assessment (RA) of NMs and advanced materials faces many challenges due to the diversity of materials and their dynamic nature. Therefore, new approaches and tools are being developed to support the assessment of NMs. As with chemicals RA generally, a strong focus is placed on New Approach Methodologies (NAMs), innovative *in vitro* and *in silico* scientific models, methods and technologies to improve safety and RA, particularly as alternatives to traditional animal testing. NAMs include *in silico* models, *in vitro* approaches based on cell cultures, high-throughput screening, and advanced bioinformatics tools. These methodologies aim to provide more human-relevant data, improve efficiency in RA, reduce costs, and address ethical concerns by minimising the use of animals in research [8], [9]. The development of NAMs for the RA of nanoscale and advanced materials is driven by several key factors. Ethical considerations, such as reducing animal testing, play a significant role, alongside scientific advances that offer more human-relevant data through innovative technologies such as advanced cell culture models and computational modelling. Regulatory and policy drivers, including legislative mandates and the push for global harmonisation, further encourage the adoption of NAMs. Technological innovations, including advances in biotechnology and computational power, enhance the precision and efficiency of NMs safety data generation. Economic factors also favour NAMs due to their cost-effectiveness and faster processing times. Additionally, the public and industry demand for ethically produced products and corporate responsibility initiatives support the transition to NAMs. NAMs are thus poised to revolutionise RA, supporting the development of safer, more ethical, and scientifically advanced approaches to NMs safety and risk assessment [10]. However, acceptance of NAMs in RA has so far been limited and they are currently validated against *in vivo* methods, despite not measuring similar end-points, a fact that is increasingly recognised by policy makers [11].

To address these challenges, nanoinformatics has emerged as an interdisciplinary field that intersects nanotechnology and informatics. It focuses on the management, manipulation, and analysis of nanoscale data with the aim to drive innovation in nanotechnology and related fields. Nanoinformatics integrates data-centric and physics-based approaches to address complex challenges in nanotechnology and nanoscience, encompassing the development and utilisation of computational tools, databases, and methodologies to optimise the synthesis, characterisation, and application of NMs. Utilising computational tools to forecast the properties and the toxicity of NMs, and indeed the development of fully *in silico* NMs whose properties can be systematically varied and used as inputs to models to explore toxicity, will enhance material safety through application of the SbD principles prior to lab-scale or industrial-scale production [12]. For example, predicting the interactions of nanoparticles (NPs) with biological systems enables designers to engineer NPs with specific surface characteristics that optimise performance and minimise potential risks [13], [14], [15]. Systems biology approaches and the integration of omics data are pivotal in understanding the complex interactions of NMs with biological systems, providing insights into the molecular mechanisms underlying NM toxicity, which is crucial for developing predictive models that are both accurate and applicable in real-world scenarios [16]. These various predictive models enhance the development of NMs by reducing the need for extensive physical testing, thereby accelerating innovation. Examples include using Quantitative Structure-Activity Relationship (QSAR) models to predict toxicity and employing machine learning (ML) models to ascertain the viscosity of nanofluids, which are critical for optimising fluid properties. These approaches reduce the reliance on extensive experimental protocols, using existing datasets to predict properties of untested NMs and employing high-throughput screening to rapidly assess numerous NMs simultaneously [16], [17].

Data management and adherence to community and regulatory standards, for both experimental and computational data generation, plays a crucial role in facilitating this predictive approach. Examples of community standards in the nanosafety areas include the MIRIBEL (Minimum Information Reporting in Bio–Nano Experimental Literature) [18] and MINBE (Minimum information about Nanomaterial Biocorona Experiments) [19] for experimental data, while MODA (Modelling Data) [20], [21] is an example of a community standard for modelling. Examples of regulatory standards include the OECD Test Guidelines for chemical testing [22] and the OECD guidance for validation of Integrated Approaches to Testing and Assessment (IATA) [23]. The implementation of standardised data formats, such as ISATab nano [24], identifiers such as nano-InChI [25], and adherence to the FAIR (Findable, Accessible, Interoperable, Reusable) data principles ensure that NM data are effectively shared and can be reused across platforms, which is essential for developing global safety standards and fostering collaborative research [9], [13]. Recently, bespoke databases for nanoinformatics have begun to emerge, including NanoPharos (https://pharos.novamechanics.com/nanopharos.html), as well as platforms for nanoinformatics analysis such as Enolas, Isalos, Jaqpot, VINAS [26] and others [27], [28]. Significant efforts have been made to curate and homogenise transcriptomics data in nanosafety [27], highlighting also the need to extend the FAIR principles to include important considerations on the experimental design and the computational methods used to analyse toxicogenomics data [28]. Large collections of toxicogenomics data have recently been modelled enabling discovery of a common regulatory mechanism of response to NM exposure in multiple species, hence defining the first One Health model of nanoparticulate exposure [39]. Omics data analysis traditionally aimed to distil lists of molecules that are statistically significantly altered by the exposure as compared to the negative controls. However, lists of molecules do not explain the complex patterns of molecular interactions and regulations that occur in biological systems adapting to dynamic environments. Network models can be used to highlight molecular circuits that occur in multiple biological systems responding to the same NM exposures [61]. Toxicogenomics-based evidence is not yet completely included in regulatory evaluations. One possibility to speed up this process is to systematically map toxicogenomics data onto Key Events (KE) of Adverse Outcome Pathways (AOPs). This approach can return an easy to interpret fingerprint of the NM mechanism of action and can facilitate the identification of biomarkers to be screened in a high-throughput fashion [30], [31].

The concepts of SbD and "Safe and Sustainable by Design" (SSbD) for NMs have also emerged as critical strategies. SbD emphasises integrating safety considerations early in the design phase of NMs, aiming to mitigate adverse effects on human health and the environment. This strategy is essential to avoid the costly and complex mitigation required if hazards are only identified after a product reaches the market. Similar considerations apply for early sustainability assessment, which advocates for use of green chemistry approaches and a focus on circularity (re-use, recycling) at the design stage. Implementing SbD and SSbD can facilitate compliance with increasingly stringent regulations concerning NMs, potentially reducing time to market and minimising regulatory hurdles [29], [32], [33]. Challenges in standardising and harmonising methods and protocols for NM testing and data reporting across different jurisdictions remain prominent. The limited availability and size of datasets for NMs pose significant challenges for data-driven approaches in safety assessments and regulatory frameworks. There is a growing need to develop and validate RA models that integrate NM exposure and hazard data to accurately predict risks associated with NMs. Finally, there is also an increasing interest in integrating NAMs, including alternative testing strategies and computational modelling, into regulatory frameworks to minimise whole animal testing and provide a mechanistic understanding of NM (eco)toxicity [34].

**Fig. 1:**
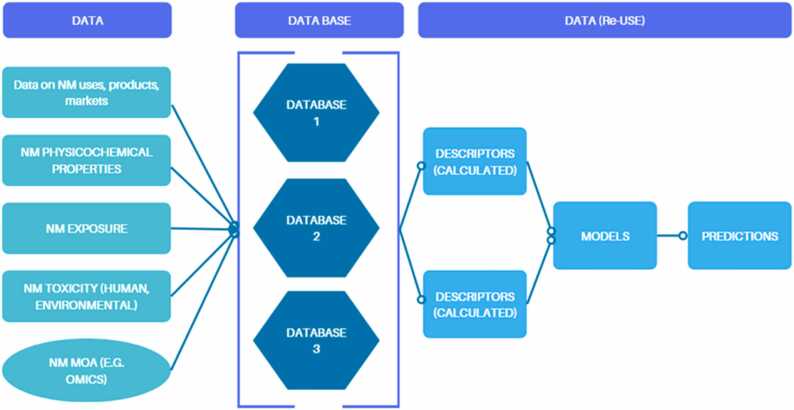
Simplified Data Flow proposed in the EU-US Nanoinformatics 2030 Roadmap.

The EU-US Nanoinformatics 2030 roadmap [34] suggested a clear data flow whereby data on NM usage, properties, exposure, and toxicity are federated in databases, increasing data availability and facilitating access as shown in Fig. 1. Descriptors derived from this data inform computational models that predict NM behaviours crucial for RAs. This structured approach promotes the "SbD" strategy (and is easily extensible to SSbD which emerged more recently), anticipating risks early in the design process. Ultimately, this strategy could standardise NM data use, support new testing methods, reduce animal testing, and improve safety and regulatory compliance in nanotechnology [34], and is the basis of the CompSafeNano apporach. The CompSafeNano Project adopts this simplified data flow, emphasising an integrated and systematic approach to NM data collection, processing, and application in modelling and SbD/SSbD.

## 2 Project description

### 2.1 Scope, objectives, and Project structure

The CompSafeNano project supports the development of inherently safer NMs by adopting the SbD principles and leveraging the combined capabilities of established nanoinformatics frameworks such as NanoSolveIT and NanoCommons, as well as governance project RiskGONE and data interoperability-focused project WorldFAIR. This holistic approach aims to close the methodological gaps highlighted by the EU-US Nanoinformatics Roadmap [34], fostering innovative collaborations that bridge gaps across different knowledge domains. CompSafeNano is dedicated to fostering innovation through a synergistic exchange of knowledge, underpinned by a strategic plan for collaborative research and staff exchanges among premier institutions across the EU and globally. This effort not only ensures the wide dissemination of scientific findings but also their effective implementation to shape safety standards and influence regulatory frameworks globally. The project's integrative approach seeks to consolidate methodologies, broaden interdisciplinary learning, and build a comprehensive knowledge base for NMs SbD and nanoinformatics innovation. A key objective is the training of potential end-users, including SMEs, industry leaders, regulatory bodies, and research scientists, achieved through an array of training materials such as detailed tutorials, documentation, videos, and specially organised information sessions, coupled with the CompSafeNano focus on providing user-friendly web applications and graphnical user interfaces (GUIs) for all of its computational models. These educational efforts are aimed at enhancing understanding of advanced nanoinformatics techniques for predicting the potential risks of NMs and comprehending their environmental impacts throughout their life cycle.

**Fig. 2:**
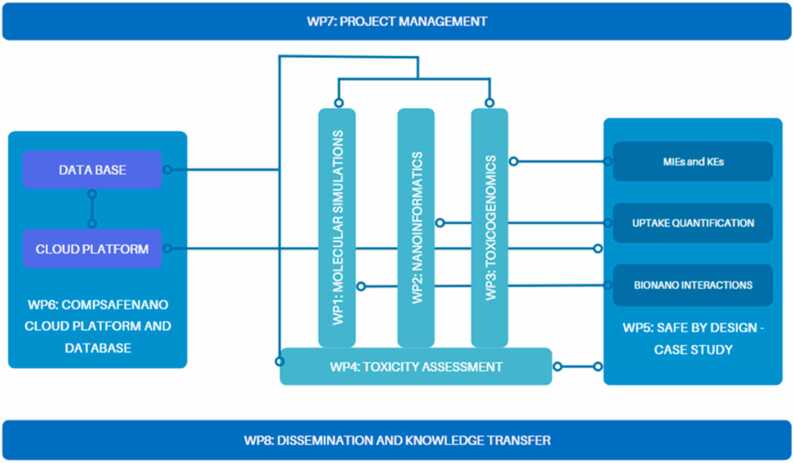
CompSafeNano WP structure and WP interactions, interdependencies and multidisciplinary aspects.

CompSafeNano is structured into 6 technical and 2 supporting WPs as shown schematically in Fig. 2. The WPs integrate all partners and are driven by the staff exchange (via secondments) that underpin the research activity of CompSafeNano and collectively build towards a toolbox for *in silico* SbD and SSbD. Descriptors for NMs generated in WP1 are fundamental to the SbD and SSbD strategies in WP5: the bionano-interaction descriptors and molecular properties from WP1 are directly fed into the development and refinement of NMs and their life cycle Assessment (LCA) in WP5. Results from WP2, including the artificial intelligence (AI) and machine learning (ML) models being developed, utilise the toxicogenomics data generated in WP3 to predict the toxicity of NMs, while ML-based image analysis techniques are used to support the assay optimisation in WP4. Moreover, the data-driven models from WP2, in combination with the predictive models from WP3, will be applied in WP5 to ensure that SbD NMs do not trigger molecular initiating events (MIEs) or key events (KEs) in the identified toxicological pathways. The assays optimised for regulatory endpoints in WP4 will be applied in WP5 to test and validate the safety of NMs designed according to the SbD and SSbD principles. Furthermore, all computational datasets developed in WP1, all omics data curated, all models developed in WP2 and WP3, standard operating procedures (SOPs) and optimised assays developed in WP4, and SbD intervention points and LCA data generated in WP5 will be documented and hosted on the CompSafeNano Cloud Platform in WP6 and in the NanoPharos database that is optimised for nanoinformatics modelling. Finally, the case studies in WP5, that will demonstrate the regulatory readiness of models and tools, utilising the cloud platform and database resources developed in WP6, will be integral to the training and dissemination activities in WP8. WP7 is essential for coordinating the efforts of all WPs, ensuring that tasks are carried out efficiently and synergies between WPs are maximised.

### 2.2 Methodology

**Fig. 3:**
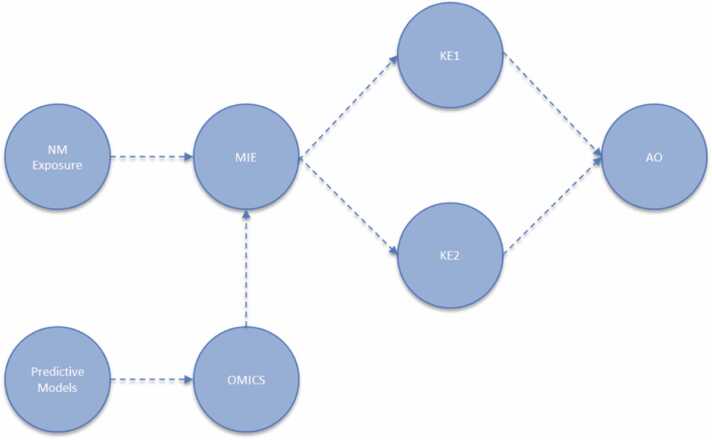
Schematic illustration of a network of MIEs, KEs, and AOs relevant to NMs, highlighting the role of omics data and toxicogenomics in mapping these pathways and demonstrating how predictive models are used to understand the interactions and potential adverse effects of NMs.

**Computational Materials Modelling:** At the core of CompSafeNano's methodology are sophisticated quantum mechanical and atomistic methods, such as Density Functional Theory (DFT) and *ab initio* Hartree-Fock (HF), which calculate properties for NMs containing up to 40 atoms. For larger NMs approaching sizes of 5 nm, semi-empirical methods like PM6 and PM7 (parameterised quantum mechanical methods used for molecular simulations) are utilised. These computational techniques are vital for generating nano-specific quantitative and qualitative descriptors that are integrated into multi-scale predictive modelling [35]. At the larger scale, the NM intrinsic and extrinsic descriptors and bionano interactions are addressed with atomistic molecular dynamics simulations, which use existing force fields. This methodology extends to developing force-fields and next-level model complexity, which includes mesoscale and continuum models, to predict NM corona formation, cellular attachment, uptake, biokinetics, and biodynamics of NMs [36], [37]. These models feed into the development of Adverse Outcome Pathways (AOPs) by addressing potential MIEs and KEs (Fig. 3). Furthermore, the computational characterisation already performed is used to train models to predict bionano interactions for hitherto unseen molecules and materials via read-across type approaches [38]

**Structure-Property/Toxicity Relationships via AI and ML:** CompSafeNano incorporates AI and ML to develop structure-property and toxicity relationships for NMs. This approach includes toxicogenomics to understand the molecular mechanisms induced by NMs. By monitoring cellular perturbations and shifts in gene expression, the project can assess the toxicity endpoints at a high throughput and relate them to the NM descriptors [39]. This bioinformatics approach helps to inform safer NM designs and supports the development of standards for data generation, collection, pre-processing, modelling, and interpretation.

**Advanced*****in vitro*****models; Alternative *In Vitro* Toxicity Assessment and Regulatory Readiness:** NAMs are developed for different exposure routes and different target organs, adjusting the test systems to better mimic human biology. The acceptance of NAMs in RA is so far limited. Formal guidelines and validation of methods and models are urgently needed. To bridge the gap between current nanosafety science and regulatory needs, CompSafeNano is pioneering the development of tiered, optimised *in vitro* assays, utilising standardised and pre-validated advanced lung and liver 3D models. Regulatory-relevant genotoxicity endpoints for these models include combined micronucleus and comet assays, which can be coupled with omics analysis and ML-based imaged analysis. These assays are designed to emulate *in vivo* responses accurately, and are structured across different biological levels to validate AOPs and boost regulatory confidence in nanoinformatics approaches.

**Integration of Materials Modelling, ML, and Toxicogenomics for RA:** CompSafeNano is dedicated to harmonising materials modelling, ML, and toxicogenomics into a unified RA strategy. Central to this initiative is the CompSafeNano e-platform, an advanced framework that not only hosts, curates, and annotates NMs data but also integrates and operationalises models and methodologies through user-friendly web services that demoncratise access to advanced nanoinformatics approaches as users do not need to have coding skills in order to implement the models themselves. This integrated system is designed to bolster decision-making and enhance the RA capabilities for NMs, through detailed documentation of the models themselves (via the MODA templates for exmaple) and the underpinning satasets, and by ensuring comprehensive support throughout the decision-making process whether that be for SbD or RA**.**

**Evolving Regulatory Framework:** CompSafeNano aligns with the latest regulatory frameworks, including REACH amendments specific to nanoforms. It strives to ensure that the models and tools developed are supportive of these regulations, facilitating the inclusion of *in silico* approaches and NAMs into regulatory assessments.

**Nature of NMs for Computational Activities:** The project utilises a diverse set of NMs, ranging from carbon-based materials to metals/metal oxides and nanocomposites, including real (commercial) materials and their *in silico* alternatives via which very precise systematic various of NMs properties can be made, to identify thresholds above or below which specific toxicological effects manifest for example. The selection and testing of these materials are coordinated closely with project partners, ensuring the use of high-quality materials and the integration of extensive experimental and *in silico* data sets from previous research initiatives.

### 2.3 Consortium and funding source

**Fig. 4:**
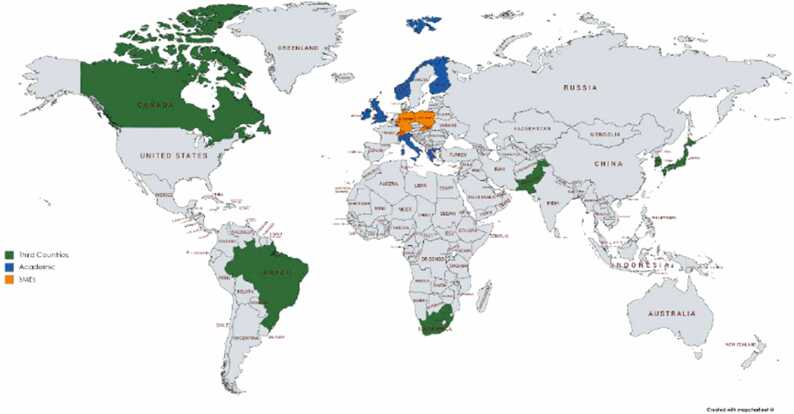
Geographical distribution of CompSafeNano partners.

The CompSafeNano consortium is a carefully assembled team, combining 22 diverse partners from across the globe, including 9 European academic institutions, 7 non-academic organisations (primarily SMEs), and 6 international academic entities, with broad geographic representation spanning 13 EU countries along with a significant international presence (see map in Fig. 4).

This consortium was strategically chosen to balance expertise across multiple fields relevant to nanoinformatics and nanosafety. Expertise within the consortium extends from chemistry and materials science to engineering, physical sciences, environmental and biological sciences. This is augmented by specialised knowledge in bioinformatics, big data analytics, computational sciences, and software and hardware development. Additionally, the partners possess skills in ontology development, knowledge management, RA, innovation management, NMs synthesis, nanotoxicology, Life Cycle Assessment (LCA) and SbD/SSbD best practices.

Operationally, the CompSafeNano consortium are interconnected with major ongoing or completed projects and initiatives that define the field of nanosafety and nanoinformatics. CompSafeNano builds directly from previous projects such as RiskGONE, NanoSolveIT, NanoCommons, SABYDOMA, and is feeding into newer initiatives such as DIAGONAL, INSIGHT, CHIASMA and PINK. Such connections enhance the project's capacity to draw on past learnings and integrate them into the current project's scope, driving innovation and ensuring the development of practical, scientifically backed solutions.

A significant focus of the CompSafeNano consortium is on validating and benchmarking the tools developed in the project to enhance their regulatory readiness. This is facilitated by the active roles played by many CompSafeNano partners in standardisation and harmonisation activities at the OECD level, as well as collaborating with CEN ISO, VAMAS, CoDATA, IUPAC and other standardisation organisations. By engaging directly with industry and regulators, the consortium aims to facilitate the widespread adoption of these innovations, ensuring they can effectively inform regulatory decision-making processes.

The CompSafeNano project is funded by the European Union’s Horizon 2020 research and innovation programme under the Marie Skłodowska-Curie grant agreement No 101008099.

## 3 Impact

### 3.1 Nanoinformatics approaches

One of the results from collaboration between several H2020 projects including CompSafeNano was a protocol for development of harmonised data reporting templates to consistently capture data and metadata. The harmonised templates are supported by the Template Wizard - a tool developed to help researchers create machine-readable templates, based on extensive experience in nanosafety data collection and aligned with community standards [40]. The Template Wizard includes more than 80 templates for various assessments of NMs and has been extended for microplastics and advanced materials research. The harmonised templates enhance interlaboratory comparisons, data reuse, and meta-analyses, aiding in the safety evaluation and regulation of nano and advanced materials [40].

From the resulting harmonised data repository and others, CompSafeNano re-uses the data as a basis for calculating critical descriptors using advanced computational and atomistic models, alongside AI and ML. The predictive power of statistical models of nanotoxicity relies heavily on the availability of relevant material descriptors, such as reactivity and interfacial properties. Given that these critical properties are not always routinely measured but can be calculated, the CompSafeNano project aims to address these gaps using advanced computer simulations and innovative computational tools, with three of the tools developed thus far described in this section.

### 3.2 Advances in NM descriptors and prediction of bionano interactions

Significant advances were made in generating atomistic/quantum-mechanical (QM) descriptors for NMs. Over 60 atomistic nanodescriptors were evaluated for more than 50 different NMs, with calculations performed in 1-nm steps for materials ranging in diameter from 2 to 80 nm. These NMs included a variety of metals and metal oxides such as Ag, Al, Au, Co, Cu, Ni, Mo, Pd, Ti, and others, in both spherical and hexahedral shapes. The descriptors were calculated to provide detailed insights into the surface and core regions, facilitating a better understanding of the materials' properties. Additionally, the project developed a NM shell depth calculator based on the Kneedle algorithm to optimise the (notional) outer shell depth for different atoms, significantly enhancing the precision of these models. The project also focused on evaluating protein-NM interaction energies for a vast number of proteins (over 240,000) from the AlphaFold database with more than 360 NMs. Coarse-grained models for interactions involving polyethylene-acrylic acid (PE-AA) and graphene oxide (GO) with tannic acid (TA) and atrazine (ATZ) were developed, which are critical for understanding the complex interactions at the bionano interface. Moreover, the project created corona-based bionano interaction fingerprints specifically for NM activity in *Daphnia magna* and developed ML-based models for small molecule interaction potentials with NMs. These comprehensive efforts culminated in the calculation of 20,274 bionano interaction potentials, providing a robust dataset for further predictive modelling and safety assessment. Publications are in preparation currently.

#### 3.2.1 Mathematical description of *in vivo* NP delivery

**Fig. 5:**
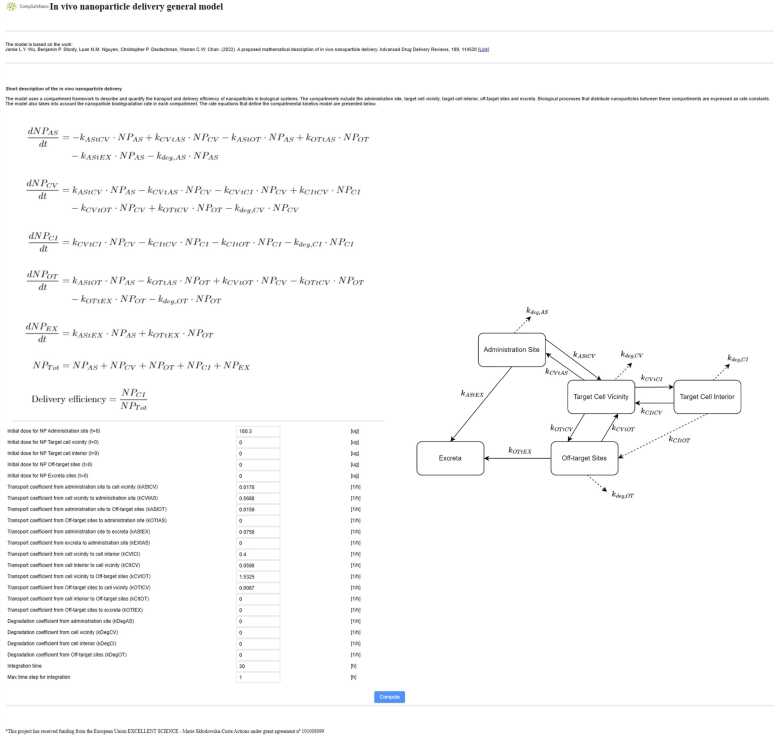
The *in vivo* NP delivery general model GUI on the CompSafeNano Cloud Platform, illustrating the kinetics of NP administration and distribution among various physiological compartments, including administration site, target cell vicinity, target cell interior, and off-target sites.

One of the models developed during the CompSafeNano project integrates compartmental kinetics to simulate the *in vivo* behaviour of nanomedicines, focusing on targeted delivery systems. The model’s technical framework involves advanced computational tools to predict and analyse the behaviour and interaction of NMs within biological systems, to identify potential toxicological effects and optimise NM design to enhance their safety profile before they are synthesised and used in various applications. The developed kinetic model involves reversible transport between five compartments related to drug delivery, specifically the administration site, off-target sites, target cell vicinity, target cell interior and excreta, as shown schematically in Fig. 5. The model developed is an extension of the conceptual model proposed by Wu *et al*. [41].

### 3.3 UANanoDock

**Fig. 6:**
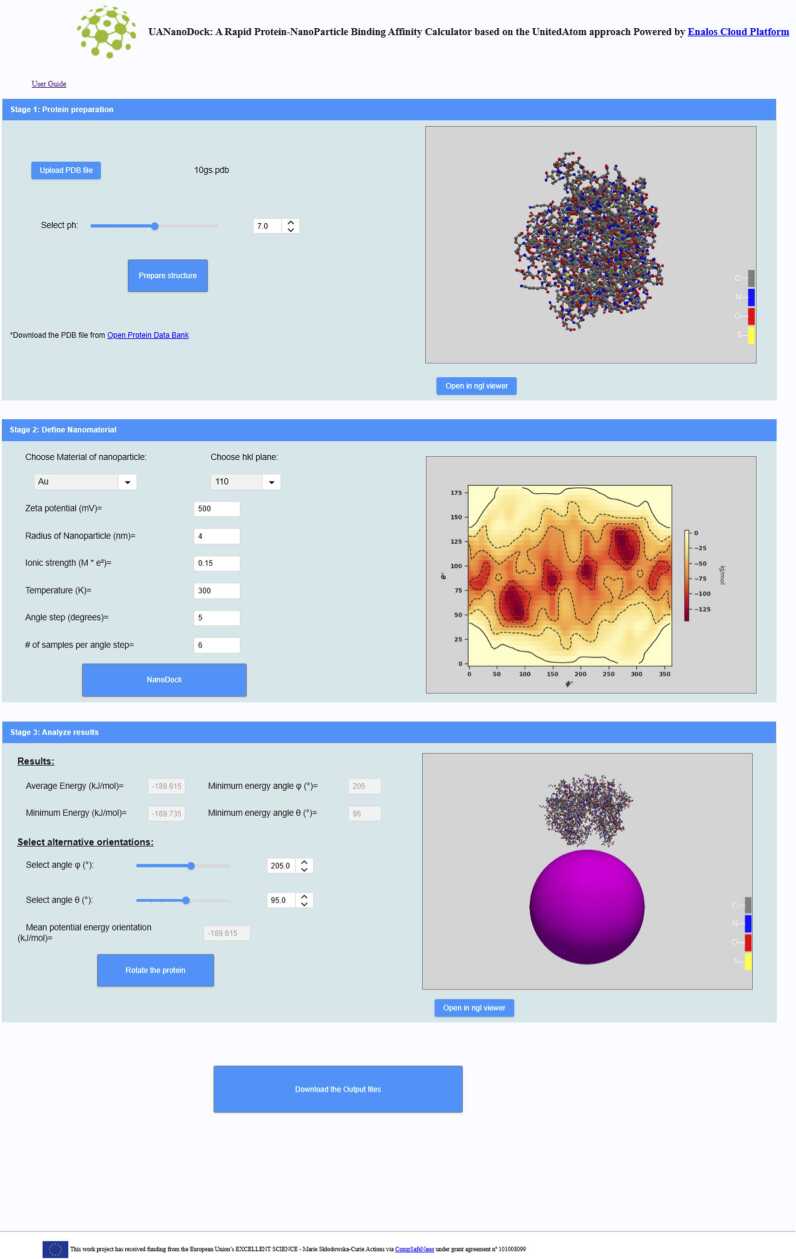
The UANanoDock GUI hosted on the CompSafeNano Cloud Platform depicting the protein preparation stage of the tool.

Another tool developed within CompSafeNano is the UANanoDock web tool. This web-based application, accessible through a user-friendly GUI via the CompSafeNano Cloud Platform, leverages a United Atom multiscale modelling approach to predict how proteins adsorb onto NMs, as shown in Fig. 6 [42]. The core functionality of UANanoDock involves simulating the interaction between proteins and NMs by computing adsorption energies and determining favourable orientations of proteins on the NM surface. This is achieved through simulation of the protein’s amino acids in their preferred ionisation states at varying pH levels, against the NM’s surface properties such as material type, size, and surface potential. An example application of UANanoDock is the analysis of the interaction dynamics between the Immunoglobulin G (IgG) antibody and spherical NMs such as AuNPs. The tool effectively maps out the preferred binding sites and orientations of the IgG antibody across NMs of different sizes, providing crucial insights that are instrumental in understanding the interfacial behaviour of proteins with NMs. UANanoDock enhances NM safety by predicting interactions between NPs and biological molecules. Its integration into CompSafeNano's toolkit supports the project's goal to minimise health and environmental risks, boosting the development of predictive models for safer NMs.

## 4 Compatibility between the next-generation risk assessment (NGRA) approach and current nanoinformatics methodologies

The CompSafeNano project partners conducted a thorough assessment of existing nanoinformatics models to determine their compatibility with Next-Generation Risk Assessment (NGRA) principles, which prioritise safety and are driven by exposure and hypothesis-based approaches. The evaluation emphasised the importance of incorporating NAMs, which include sophisticated *in vitro* techniques, comprehensive omics analyses, and advanced computational modelling into NGRA. These methodologies are crucial for enhancing the understanding of NMs' properties and their interactions with biological systems [43]. The project highlighted the integration of diverse data sources and the extrapolation of *in vitro* mechanistic insights to *in vivo* outcomes, to develop robust, predictive models that align with the NGRA’s focus on addressing adverse outcomes rather than merely identifying perturbations [44].

The CompSafeNano project extensively discussed the recent advances and persistent challenges associated with *in vitro* to *in vivo* extrapolation (IVIVE) models, particularly focusing on the critical task of extrapolating *in vitro* doses to relevant *in vivo* responses. The project underscored the necessity of accurate dose extrapolation, emphasising the integration of kinetic information to enhance the reliability and predictive power of these models [45]. Additionally, the AOP methodology, which is instrumental in identifying mechanistically relevant events that serve as biomarkers for targeted *in vitro* assays, was also considered. However, the project noted the limited availability of AOPs specific to NMs and highlighted ongoing efforts to develop nano-specific AOPs to better understand the potential adverse effects of NMs [46]. The CompSafeNano project reviewed the development and application of QSAR models for predicting the toxicity of NMs. The review encompassed perspectives on QSAR models tailored for various biological systems, including respiratory, dermal, gastrointestinal, and immunological systems. The project emphasized the critical need for improved data integration to enhance the robustness of these models [47]. Additionally, it highlighted the necessity for developing more sophisticated QSAR models that can accurately predict NM toxicity across different biological contexts, ultimately contributing to more reliable and comprehensive nanosafety assessments [48].

A key challenge in all model development is the underpinning data quality - CompSafeNano partners also contributed to a review of the potential NMs interferences in common *in vitro* assays and appropriate controls to test for such interferences [49].

The CompSafeNano project identified several key challenges, including the proper selection of *in vitro* assays (and the need to check for NM interference in the selected assays), accurate dose extrapolation, and the development of comprehensive predictive models. To address these, the project outlined future steps such as refining *in vitro - in vivo* exptrapolation (IVIVE) models, enhancing data integration, and increasing the predictive accuracy and applicability of nanoinformatics models [46], [47]. The review also discussed the use of QSAR models in regulatory-relevant safety assessments, emphasising the need for standardised experimental procedures and robust data management to ensure reproducibility and reliability[50]. Additionally, the project developed strategies for assessing the ecotoxicity of NMs and integrating these data into predictive models. Planned experiments aim to understand the behaviour, properties, and uptake of NMs in various environmental contexts, thereby contributing to a comprehensive understanding of their potential ecological impact [51].

## 5 Toxicogenomics modelling and omics data

The CompSafeNano project has also made significant progress in the domain of toxicogenomics modelling and the utilisation of omics data, aiming to develop predictive models and mechanistic insights into the behaviour of NMs. One of the primary achievements includes the ongoing curation of public toxicogenomics datasets, which encompasses a variety of NMs such as 2D materials (e.g., Graphene, MXenes), NMs used in pesticides, and silica NMs in the development of toxicogenomics-based predictive models; the project has successfully utilised these curated datasets to create models that predict MIEs and AOPs from toxicogenomics data. For instance, predictive models for ecotoxicological endpoints from toxicogenomics data were developed, and the creation of Quantitative Structure-Mutation-Activity Relationship Tests (QSMART) models was achieved. These models are instrumental in elucidating the mechanisms of action (MOA) of NMs and for identifying predictive biomarkers from multi-omics data, which are crucial for comprehensive RAs.

## 6 Αdvancing the regulatory readiness levels of the advanced models and in *vitro* methods

One of the main aims of the CompSafeNano project is to consolidate the developments achieved during the project, either from academia or industry, in the domain of alternative *in vitro* models. These developments include: a) Further refinement of existing protocols and stnadard operating procedures (SOPs), aiming at increasing the throughput, reproducibility and transferability of the methods as well as their reliability; b) training of staff on the use of the *in vitro* models; c) inclusion of additional biological readouts and refinement of existing ones; d) technology and knowledge transfer of the technologies for hazard and RA; e) expansion of the applicability domains of the alternative *in vitro* testing systems to new domains (e.g., new materials, new applications, etc.); and f) further development of advanced *in vitro* 3D models, micro-tissues and mini-organs (e.g., lung and liver co-culture models, etc.) coupled with regulatory relevant endpoints for cytotoxicity, genotoxicity and other critical endpoints [52]. All the aforementioned activities are focused particularly on advancing the regulatory readiness levels of the *in vitro* methods and on how to foster their regulatory applicability. Among the different test systems that are being further developed, are *in vitro* inhalation methods which have been tested on several new materials and particles from CompSafeNano (e.g., graphene oxide (GO) from Danubia NanoTech, or functionalised PLGA-gold NPs from MyBiotech). In addition to advanced *in vitro* models and inhalation toxicity assessment, other *in vitro* tests have been performed using cytotoxicity tests such as Alamar Blue or colony forming efficiency (CFE) assays and other eco-toxicological models. This has allowed further characterisation of the applicability domains of the *in vitro* models for assessment of new advanced materials and, thus, to increase the potential market uptake for the *in vitro* methodologies. This will also enable the generation of new experimental data that can be used to further develop and train *in silico* methods for toxicity prediction and grouping/read-across.

Focus has been given to the training of research staff on *in vitro* methods for genotoxicity, which is a critical regulatory relevant biological outcome that needs to be addressed for RA under the REACH regulations and as part of the Classification and Labelling of Products (CLP) regulations and which, typically relies on and, is performed using, *in vivo* methods. Following training in the CompSafeNano-agreed protocols, several partners have worked to integrate Comet and micronucleus assays into their advanced *in vitro* models, thus increasing the relevance and reliability of these NAMs. Most importantly, as part of the CompSafeNano project, a comprehensive review has been conducted (currently under consideration in Mutation Research) assessing the suitability of NAMs for detecting the genotoxicity of nanoscale and advanced materials and discussing the barriers to their regulatory acceptance. Extensive OMICS studies (e.g., from MACRAME and SCENARIOS) are being carried out using different classes of chemicals (e.g., graphene, PFAS, respiratory irritants, respiratory sensitisers, etc.) with the aim of further characterising the models and comparing the analytical power of different -OMICS techniques, including comparing bulk transcriptomics to single cell transcriptomics. This exercise will also demonstrate that the *in vitro* methods are able to pick up the relevant MIE and KE and are thus suitable for AOPs-based approaches.

### 6.1 SbD intervention points

**Fig. 7:**
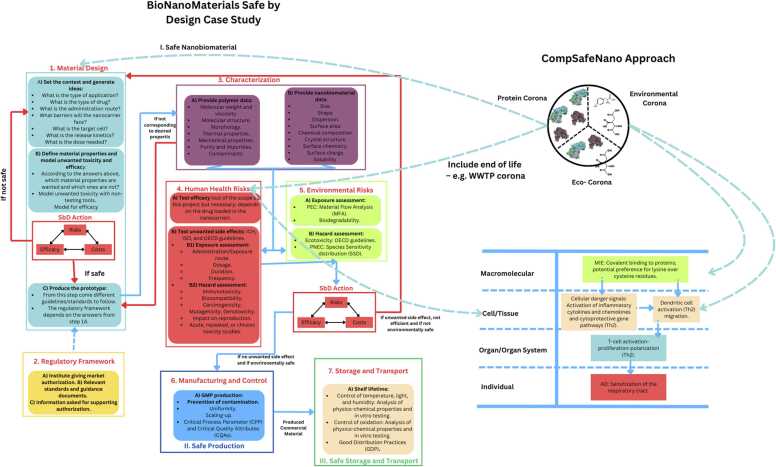
CompSafeNano extension of the nanobiomaterials for drug delivery SbD case study [53] to include also (i) considerations of the acquired biomolecule corona (both biological and environmental), the AOP framework for linking molecular initiating events and KEs to adverse outcomes, and the LCA approach to ensure that all aspects of NMs production, processing, use and disposal are covered in the inventory of SbD intervention points.

SbD intervention points, which are critical stages where safety measures can be integrated, have been systematically identified in CompSafeNano to mitigate potential risks associated with NMs, ensuring safety and sustainability throughout their lifecycle. This approach, based on literature analysis, of targeting key stages in the life cycle supports proactive risk management, regulatory compliance, and stakeholder collaboration, thereby fostering public trust in nanotechnology innovation. In developing the SbD intervention points inventory, CompSafeNano draws on significant insights from nanomedicine and biotechnology. From the nanomedicine field, a detailed methodological approach focusing on polymeric nanobiomaterials for drug delivery which ensures efficacy and safety from the initial design stages through thorough characterisation and iterative optimisation [32], [53] has been leveraged as a starting point for extension beyond the clinical setting and generalising to other materials. From biotechnology, the project adopts risk management strategies early in the design phase, incorporating measures such as physical containment, self-destruct mechanisms, and biosensors to mitigate potential risks associated with genetic engineering [54]. Integrating these insights, CompSafeNano's SbD inventory considers the acquired biomolecule corona, employs AOPs to map biological effects, and uses life cycle assessment (LCA) to evaluate environmental impacts, ensuring comprehensive safety and sustainability throughout the NMs' lifecycle, as shown schematically in Fig. 7.

The SbD intervention points identified by CompSafeNano encompass critical stages and properties throughout the lifecycle of NMs. As a starting point, during the synthesis phase, intervention points include implementing synthesis techniques that allow precise control over NM size to reduce potential toxicity, modifying surface charge to minimise non-specific interactions with biological molecules and cells, using safer chemicals and precursors to minimise toxicological risks, adjusting solubility to enhance safety and environmental compatibility, and enhancing stability to prevent degradation into harmful byproducts. Subsequently, in the processing stage, interventions involve ensuring heat resistance to prevent thermal degradation, applying anti-aggregation agents or techniques to maintain NM dispersion, ensuring chemical stability to prevent degradation and release of potentially toxic ions, carefully controlling processing temperatures to prevent alteration of NM properties, minimising mechanical stress to prevent structural damage, and implementing stringent contamination control measures to prevent impurities. For the application phase, key interventions include designing products to minimise the release of free NMs during use, enhancing the durability of nano-enabled products to reduce wear and potential NM release, ensuring biocompatibility to reduce adverse reactions, assessing and minimising environmental impact, limiting user exposure, promoting responsible end-of-life disposal or recycling options, and maintaining functional stability under expected use conditions. Finally, at the disposal stage, interventions focus on designing NMs to be biodegradable under environmental conditions, implementing strategies for recycling or safe reuse, minimising environmental persistence to prevent long-term ecological impacts, assessing and mitigating toxicity during disposal processes, properly classifying waste containing NMs to ensure appropriate handling and disposal, providing clear and accessible disposal information to end-users and waste management facilities, and designing nano-enabled products with reusability in mind to extend their lifecycle and reduce waste.

### 6.2 Cloud platform and database

CompSafeNano aims to deliver long lasting impact and sustainability beyond the project lifetime. Key to this is maximising the take-up of the new tools for materials hazard and RA systems developed, either individually or as part of the systems solution with modules selected on an as-needed basis. This will be achieved via the CompSafeNano e-platform which consists of 2 components: (i) the CompSafeNano Database for data hosting, curation, and annotation of NMs (building on the NanoCommons and NanoSolveIT knowledge base); and (ii) all methods and models developed in the project, implemented as web services, presented as a ready-to-use user-friendly interface (Cloud Platform) to guide decision making and RA of NMs.

The CompSafeNano Cloud Platform and Database are thus pivotal components of the CompSafeNano project, designed to underpin and facilitate achievement of the project's broader objectives of enhancing NMs’ safety and sustainability. The cloud platform serves as a centralised, web-based hub providing computational modelling tools and facilitating data input and prediction generation, while the CompSafeNano Database, an extension of the NanoPharos database, ensures comprehensive data management and accessibility, supporting the parameterization of predictive modelling and interdisciplinary research.

#### 6.2.1 CompSafeNano cloud platform

One of the main outcomes of the project is the CompSafeNano Cloud Platform that will be made available to the community as a cloud application accessible via the web. It builds upon existing tools (e.g., the NanoSolveIT Cloud Platform) to maximise data and tool harmonisation, to facilitate industry & regulatory acceptance and to ensure sustainability of the tools. By the end of the project, it will provide all the computational modelling tools developed during the project under a common framework and with an optimised workflow for input of data and generation of predictions in response to specific stakeholder queries. The platform's system architecture is designed to be effective, intuitive, scalable, and simple to maintain, while being open for community involvement through integration with various tools and databases, thereby creating a centralised, web-based platform that is secure, adaptable, and able to evolve to meet specific user needs.

The CompSafeNano State-of the-art IT tools for distributed applications, such as Docker and Kubernetes, are being used to integrate the CompSafeNano database and *in silico* tools with the existing RiskGONE/NanoSolveIT Cloud Platform [55] and the NanoCommons e-Infrastructure [56], to ensure sustainability and enable flexibility and more efficient management of the microservices. All services will run in tested docker containers and will be accessible through an Application Programming Interface (API) and user-friendly graphical user interface (GUI). The open architecture of the e-platform will ensure and support its sustainability and continued evolution, as it will be continuously updated with the advances in the field, by integrating emerging material informatics tools provided by project participants, and from the entire community. NGRA services will be included in the CompSafeNano platform, offering ready-to-use web implementations of structure-property/toxicity relationships via AI and ML allowing the implementation of customised solutions. Prior to this integration, a comprehensive review was conducted to evaluate the alignment of existing informatics-based tools used in nanosafety research with the assumptions of NGRA for NMs. Following the review, subsequent discussions were held to address the identified shortcomings and challenges of solutions developed so far.

**Fig. 8:**
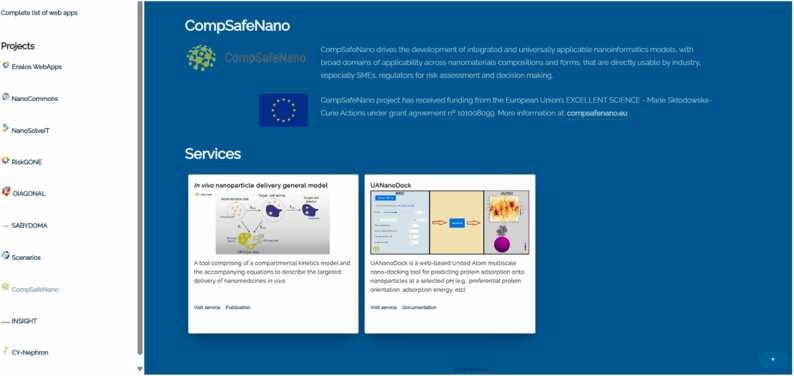
CompSafeNano Cloud Platform and its graphical user interface (GUI).

First, the recent advances in IVIVE models, which are paving the way for the future development of NGRA-oriented QSAR models, were analysed. An AOP based methodology for the identification of mechanistically relevant events that can serve as biomarkers for the targeted selection of *in vitro* assays was shown. Additionally, the challenges related to the extrapolation of *in vitro* doses into *in vivo*-relevant responses were presented. The limitations of models applied so far to study the fate of NMs in the human body, which are mainly related to limited data or knowledge available about their transformations in the biological systems in which they are applied, were analysed [45]. Additionally, the challenges and potential for improved data integration, and the importance of building consensus between the *in vitro* and QSAR domains, were discussed. Biological/toxicological experimentation has evolved significantly in recent decades, utilising diverse high-throughput techniques to capture numerous cellular and organismal measurements across various organisational levels (molecular, organelle, single cell, or tissue). Simultaneously, the capacity of *in vitro* systems to mimic human physiology has advanced considerably. Therefore, the future nano-QSAR methodology should follow modern toxicological studies and take full advantage of the opportunities offered by modern toxicological platforms [52]. Hazard and RA tools from previous projects (e.g., tools from NanoCommons, RiskGONE & NanoMILE) are already accessible as user-friendly applications and are being further developed. Many of the predictive nanoinformatics models mentioned [8], [14], [57] are also hosted on a similar Cloud Platform architecture and are publicly available. The CompSafeNano Cloud Platform [8], based on web semantic cheminformatics and nanoinformatics applications, can host any predictive model as a web service with a user-friendly interface. The currently hosted models are shown in Fig. 8 below.

#### 6.2.2 CompSafeNano Database

NanoPharos (https://db.nanopharos.eu/) is an advanced database designed to store, curate, and organise environmental health and safety (nanoEHS) data on NMs, developed during the NanoSolveIT & NanoCommons projects. It draws data from literature, experiments, and computational methods including ML to support nanoinformatics research, ensuring compliance with the FAIR principles. Features of NanoPharos include dynamic management of NMs, computational analysis, the inclusion of omics data, and the use of identifiers like the European Materials Registry (ERM) [58] and NanoInChI [25] for maintaining accuracy and supporting detailed analysis.

**Fig. 9:**
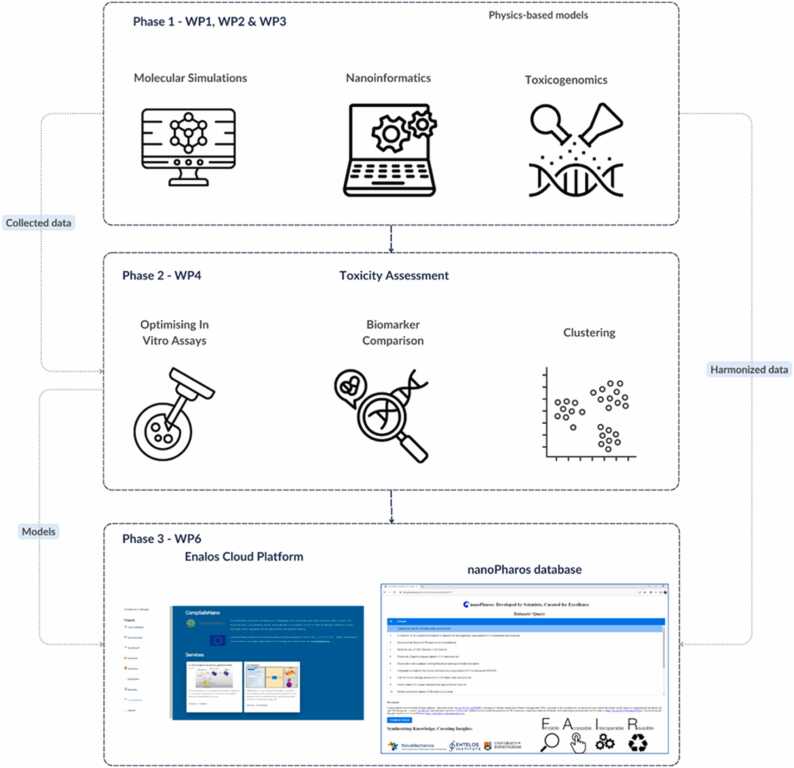
Schematic representation of the data organisation within the nanoPharos database.

The database is being further expanded within the CompSafeNano project as a primary data management solution, fully integrated through APIs with other NMs-related databases (NanoSolveIT knowledge base, NanoCommons knowledge base, eNanoMapper, etc). Accessible through a REST application programming interface (API), NanoPharos provides a modular architecture that supports FAIR data through unique identifiers, rich metadata, and user-friendly search options, as shown schematically in Fig. 9. It has been designed intentionally to support predictive modelling, facilitating interdisciplinary research, and promoting the safe development of nanotechnologies by offering open-access, harmonised data. Programmatic access via API is key to enabling integration and interoperability among the database, modelling services, and the CompSafeNano Cloud Platform.

Among the new datasets adding during CompSafeNano to date are detailed examinations of LogP measurements for noble metal NMs as a proxy for the NMs hydrophobicity (dataset ID np23), adsorption efficiency of Perfluorooctane sulfonate (PFOS) to Gold NMs under varying pH conditions (dataset ID np24), reactive oxygen stress (ROS) induction by Gold NMs in HEK293 cells (dataset ID np25), zeta potential measurements for various NMs (dataset ID np26, np27), and biodistribution of PEG-Au NMs in rats after intravenous injection (Dataset ID np28), which represents a significant enrichment of the NanoPharos database.

## 7 Discussion

The activities conducted within CompSafeNano are advancing nanoinformatics in several aspects through the development of methodologies of NM characterisation and *in silico* tools implementing these methodologies. Novel NM descriptors addressing relevant physicochemical properties have been proposed and evaluated using atomistic simulations for dozens of NMs. Several NMs including common polymers and GO have been parameterised using atomistic simulations and can now be used in predictive modelling of bionano interactions and NM toxicity as well as in designing novel composite NM and drug nanocarriers.

Rapid development in toxicogenomics is enabling characterisation of the molecular responses of a biological system upon exposure in a mechanistic fashion. However, the scientific community has only recently established universal guidelines for data management, harmonisation, pre-processing and annotation, converging to the formulation of the FAIR principles. Such principles guide researchers to generate reusable data, aiding science efforts to shift from traditional animal testing to alternative approaches. Therefore, in the past, data were generated with extremely heterogeneous procedures, resulting in poor standardisation. In fact, in the realm of omics, a large proportion of such data remains non-compliant with the FAIR principles [59] limiting their integration and exploitation [28]. A recent effort to provide a solid and user-friendly tool for data FAIRification has been developed by di Lieto and colleagues [60]. This GUI allows a semi-supervised toxicogenomics metadata curation, and also implemented a Good Laboratory Practice (GLP) module, in which all the steps of the curation are recorded in a real-time GLP report.

The power of open and integrable data has been highlighted in a landmark publication by del Giudice *et al*. [61], where the authors performed the biggest reanalysis of public toxicogenomics data to date. In detail, public transcriptomics data on NMs tested on multiple human and mouse cell types and tissues (dataset ID np2), were integrated with the aim of characterising the mechanism of action of the materials and to identify a common molecular response of the exposed biological systems. The analysis shed light on a conserved response mechanism to NM exposure (mainly mediated by regulatory regions controlled by C_2_H_2_ transcription factors) that is conserved across several biological systems and throughout the evolutionary scale [39]. Although this study was challenged by limited homogeneity in the reporting of the nanosafety experiments in which the toxicogenomics data was generated, it is potentially relevant for RA in multiple species and throughout the NMs life cycle, in full compliance with the advocated One Health principles. Moreover, these investigations highlight that the molecular responses induced by NM exposures are not driven by individual entities (genes, proteins, metabolites, etc.) working independently from each other, but by a coordinated by a network of interactions that involves several molecules acting on a plethora of cellular pathways. The NMs-induced response was distinctly different from the response induced by drug molecules [39].

A novel network toxicology framework that directly interprets the molecular response to chemicals as a chain of toxicologically relevant events has recently been formulated by del Giudice *et al.* [61], and applied to contextualise the toxicological responses with respect to unexposed systems. The mechanism of action of a comprehensive set of 31 industrially relevant NMs of different chemistries, which are representative of a range of different physicochemical properties and hazard potentials were determined by integrating classical univariate analysis of transcriptomics data with both network reconstruction of the exposed system and the AOP network built by combining all the MIEs, KEs, and Adverse Outcomes (AOs) as they are linked in the AOP-wiki. Through this multi-layer analysis, a thorough interpretation of the toxicological effects of the materials under investigation was reconstructed. Therefore, network-based methods hold the potential to significantly improve the understanding of toxicological mechanisms from a systems biology perspective and provide relevant considerations and future data-driven approaches for the hazard assessment of NMs and other advanced materials.

Despite the great interest in the potential of omics technologies to clarify mechanistic aspects of chemical exposures, toxicogenomics-derived evidence still struggles to be included into regulatory considerations. There is significant global effort to standardise the data reporting towards the FAIR standards, but additional effort needs to be taken by the scientific community to increase the readability of toxicogenomics-based models,and to democratise the use of advanced computational strategies. The activities of WP3 are focussed on developing tools and methods that are as robust and rigorous as possible while ensuring their usability by a broader set of users working in academic as well as industrial and regulatory environments. WP3 results have the potential to impact the SSbD framework by providing AOP-anchored evaluations of the molecular mechanism of action of chemicals and NMs, which can be included in the first steps of the R&D process.

A comprehensive review conducted as part of the CompSafeNano project outlined the state-of-the-art *in vitro* and *in silico* methods for detecting genotoxicity of nanoscale and advanced materials. Promising techniques involve 3D organ models representing various exposure routes and different target organs. The most advanced NAMs include skin, lung and liver models for micronucleus and comet assays, along with other organ models [62]. These methods can be utilised to address challenges in current RA approaches and pave the way for the integration of NAMs within the NGRA framework. Integration of such models with AOP-anchored toxicogenomics can provide comprehensive understanding of the mechanisms underlying toxicity, which can potentially enhance the regulatory decision-making and support the SSbD framework by enabling the design of safer, more sustainable NMs and NM-enabled products.

While significant progress has been made in the development of NAMs, prioritisation is needed for specific organs such as the lung, liver and gastrointestinal tract, with a focus on genotoxicity. Despite the regulatory agencies such as ECHA, EPA, SCCS and EFSA recognising the importance of using NAMs to predict and mitigate the risks associated with NM-induced adverse effects, they also present challenges, including the need for standardisation, validation and acceptance by regulatory authorities. As highlighted in recent survey on NAMs for NMs [62], [63] for regulatory acceptance and EU implementation, key measures include: (i) Adapting exposure scenarios for diverse NM exposure routes, (ii) Adjusting test systems to mimic human biology, (iii) Developing *in vitro* exposure protocols considering NM behaviour, (iv) Creating methods for characterising NMs in pure form and in culture medium; and (v) Using existing data and databases to support *in silico* method validation. Collaboration among scientists, regulators, industry and advocacy groups is essential to ensure the reliability and robustness of NAMs, promoting a sustainable and ethical approach to risk assessment.

### 7.1 Dissemination and communication of CompSafeNano

The CompSafeNano project has executed a detailed dissemination and communication strategy to maximise the reach and impact of its nano-informatics approaches. The project has established a strong online presence through its website (http://compsafenano.eu) and social media profiles on LinkedIn and X (formerly Twitter), which are regularly updated with project news, scientific publications, and industry events. As of the latest update, the LinkedIn profile has 95 followers, and a series of thematic posts, such as "CompSafeNano Partners" and "Secondment Notes," have been used to engage the community.

The dissemination efforts have also produced a variety of promotional materials and standardised communication templates to ensure consistent messaging. A strong commitment to open access is a cornerstone of the project, ensuring that all tools, data, and publications are freely accessible. This is facilitated through both green and gold routes, supporting the project’s transparency and broad knowledge-sharing goals.

The project has also focused on the long-term exploitation of its outputs, developing innovative tools, leveraging CompSafeNano’s research outcomes. These tools and methodologies are being integrated into new Horizon Europe projects like CHIASMA, PINK, INSIGHT, and PROPLANET, demonstrating their broad applicability and impact. Stakeholder engagement has been robust, with CompSafeNano organising and participating in several key meetings and workshops. Notable events at which CompSafeNano outputs were disseminated include the NanoSolveIT & CompSafeNano common meeting during the NanoWeek in Cyprus (20–24 June 2022), provision of training in our models at the Nanosafety Training School 2023 (15–19 May 2023), presentation of our tools and approaches the Computational Methods for Modelling Bionano Interactions CECAM workshop at University College Dublin, Ireland (26–28 June 2023), and at the Sao Paulo School for Advanced Science in Nanotechnology, Agriculture, and the Environment at CNPEM, Campinas, Brazil (3–14 July 2023), and a hands-on training session at MaterialsWeek in Cyprus in June 2024. These events have not only disseminated CompSafeNano’s knowledge but also fostered collaborations within the scientific community, promoting the adoption and integration of the project’s approaches into regulatory frameworks and industry practices.

### 7.2 Future directions

The future actions planned for the CompSafeNano project include incorporating AI and ML to develop structure-property and toxicity relationships, including toxicogenomics to understand the molecular mechanisms induced by NMs. The project will develop AI/ML models based on AOPs to predict events triggering adverse effects and models to predict the toxicity of Multicomponent NMs (MCNMs) based on effects induced by individual components. CompSafeNano partners will continue their focus on development, standardisation and validation of advanced i*n vitro* models such as lung, liver and other mini-organs and microtissues to better reflect real exposure and response from target organs. Further efforts will be made in curating and analysing relevant toxicogenomics data, optimising *in vitro* assays for regulatory-relevant endpoints, and confirming MIEs and KEs for selected AOPs. The project will also organise a workshop on SSbD in collaboration with ongoing EU projects, and will continue to collect, curate, assure quality, and harmonise *in vitro* data, and apply this for the prediction of mixture toxicity for specific substances. Additionally, interpretation of AOP-toxicogenomics models and synthesis of modified GO for further testing will be conducted. Partners will encourage the nanosafety community to use harmonised data reporting templates to increase quality in data reporting and FAIRness. The CompSafeNano Database will be expanded with new datasets, and models and tools will be implemented as web services. Knowledge transfer will be accelerated through futher parner staff exchanges (secondments) to support partners in integrating their models, ensuring effective collaboration and advanced nanosafety research.

## 8 Conclusion

The CompSafeNano project is advancing the safety and innovation of NMs by integrating nanoinformatics, computational modelling, and predictive toxicology to enable SbD for NMs. The project's focus on the SbD principles aims to create inherently safer NMs while enhancing regulatory compliance and fostering international collaboration. The project leverages data-centric approaches to optimise NM synthesis, characterisation, and application using computational approaches to identify the optimal conditions prior to undertaking lab-work. By utilising databases and computational tools, CompSafeNano enables users to predict NM properties and toxicity, reducing the need for extensive physical testing. The development of NAMs, QSAR and ML models, and their presentation as user-friendly graphical interfaces helps to democratise access to these advanced tools by industry and the research community, without the need for users to have advanced programming skills themselves. Predictive models developed in CompSafeNano are used, for example, to design NMs with specific surface characteristics for targeting applications, such as delivery of a drug to its target site of action.

Data management, innovation, standardisation and validation play a crucial role in CompSafeNano's success. The project implements standardised data formats and adheres to the FAIR data principles, ensuring effective data sharing and reuse. It also establishes a comprehensive data repository, the NanoPharos database, for reliable and accessible NM data formatted for re-use in nanoinformatics modelling. Advanced toxicology methods and models, such as 3D mini-organ and mini-tissues models, and the integration of omics data and systems biology approaches, are employed to understand NM interactions with biological systems.

CompSafeNano's potential for innovation is significant. By incorporating SSbD principles early in NM design, the project mitigates health and environmental risks. The involvement of international partners fosters a collaborative environment for data sharing and innovation. Training and skill development for researchers and professionals in nanosafety is a major output from the project - both via the directly trained researchers (via the secondments) and through our focus on development of robust training materials and documentation to support widespread utilisation of all our models and tools.

Sustainability and ethical standards are central to CompSafeNano's approach. The project emphasises environmental sustainability and ethical considerations throughout the NM lifecycle. Implementing SSbD strategies promotes responsible innovation and public trust in nanotechnology. By providing innovative tools and methodologies for safer and more sustainable NMs, the CompSafeNano project significantly advances the field of nanotechnology. Its holistic approach ensures that NMs are designed with safety and sustainability in mind, positioning it as a critical driver of innovation in the nanotechnology sector.

## CRediT authorship contribution statement

*Conceptualisation:* GM, IL, AA

*Writing – original draft:* DZ, AM, AT, LAS, GdG, AF, AS, DG, IR, JS, VL, KJ, KC, BJ, AM, AS, TP, MG, VW, DSTM, RP, NE-Y, ER-P, SM, SS, VM, PT, HS, EML, TS, A-KHO, VS, PH, MD, AGP, LCG, KR, SU, SC, TS, KT, CL, FD, MMF, EB, EM, GM, IL, AA

*Writing – review and editing:* DZ, AA, IL

*Project administration:* DZ, AA

*Funding acquisition:* GM, IL, AA

## CRediT authorship contribution statement

**Diego S. T. Martinez:** Writing – original draft. **Candida Lorusso:** Writing – original draft. **Victor Wepener:** Writing – original draft. **Kaido Tämm:** Writing – original draft. **Muhammad Moazam Fraz:** Writing – original draft. **Naouale El Yamani:** Writing – original draft. **Iseult Lynch:** Conceptualization, Visualization, Writing – original draft, Writing – review & editing. **Romana Petry:** Writing – original draft. **Francesco Dondero:** Writing – original draft. **Sivakumar Murugadoss:** Writing – original draft. **Angelos Mavrogiorgis:** Writing – original draft. **Dimitrios Zouraris:** Conceptualization, Project administration, Visualization, Writing – original draft, Writing – review & editing. **Elise Rundén-Pran:** Writing – original draft. **Antreas Afantitis:** Conceptualization, Funding acquisition, Project administration, Writing – original draft, Writing – review & editing. **Vasileios Minadakis:** Writing – original draft. **Laura Aliisa Saarimäki:** Writing – original draft. **Sergey Shaposhnikov:** Writing – original draft. **Andreas Tsoumanis:** Visualization, Writing – original draft. **Sami Ullah:** Writing – original draft. **Katie Reilly:** Writing – original draft. **Mary Gulumian:** Writing – original draft. **Tommaso Serchi:** Writing – original draft. **Tomasz Puzyn:** Writing – original draft. **Sebastien Cambier:** Writing – original draft. **Anita Sosnowska:** Writing – original draft. **Ian Rouse:** Writing – original draft. **Ann-Karin Hardie Olsen:** Writing – original draft. **Dario Greco:** Writing – original draft. **Tanima SenGupta:** Writing – original draft. **Peter Hutar:** Writing – original draft. **Vladimir Lobaskin:** Writing – original draft. **Julia Subbotina:** Writing – original draft. **Viera Skakalova:** Writing – original draft. **Krzesimir Ciura:** Writing – original draft. **Anastasios G. Papadiamantis:** Writing – original draft. **Karolina Jagiello:** Writing – original draft. **Maria Dusinska:** Writing – original draft. **Alicja Mikolajczyk:** Writing – original draft. **Beata Judzinska:** Writing – original draft. **L. Cristiana Gheorghe:** Writing – original draft. **Periklis Tsiros:** Writing – original draft. **Eleonora Marta Longhin:** Writing – original draft. **Angela Serra:** Writing – original draft. **Harry Sarimveis:** Writing – original draft. **Antonio Federico:** Writing – original draft. **Giusy del Giudice:** Writing – original draft. **Georgia Melagraki:** Conceptualization, Funding acquisition, Visualization, Writing – original draft. **Emilie Brun:** Writing - original draft. **Evangelos Melagrakis:** Writing - original draft.

## Declaration of Competing Interest

DZ, AM, AT and AA are affiliated with NovaMechanics, a cheminformatics and materials informatics company; KJ, KC, BJ, AM, AS and TP are affiliated with QSAR Labs, a cheminformatics company; SS is affiliated with NorGenoTech a company that specializes in genotoxicity testing; VS and PH are affiliated with Danubia Nanotech, a company that specializes in the production and applications of carbon-based nanomaterials. EM is affiliated with the company Calculus IKE which is focused on standardisation.
